# Isolation of a clonal lineage of IgA broadly neutralizing antibodies from a chronically infected Tanzanian subject

**DOI:** 10.1186/1742-4690-9-S2-O35

**Published:** 2012-09-13

**Authors:** M Moody, MS Drinker, TC Gurley, JD Amos, JA Eudailey, LC Armand, R Parks, ES Gray, L Morris, A Finzi, X Yang, J Sodroski, H Liao, GD Tomaras, DC Montefiori, BF Haynes

**Affiliations:** 1 Duke University Medical Center, Durham, NC, USA; 2 National Institute for Communicable Disease, Johannesburg, South Africa; 3 Dana-Farber Cancer Institute, Boston, MA, USA

## Background

Only ~20% of HIV-1-infected subjects develop broadly neutralizing antibodies (bnAbs) and the origins of such antibodies remains obscure. To date, all isolated bnAbs have been of the IgG isotype.

## Methods

Memory B cells from a chronically infected Tanzanian subject with plasma broad neutralizing activity were labeled with a consensus C envelope (Env) and Env+ cells sorted as single cells. Immunoglobulin heavy and light chain genes were amplified by PCR and analyzed for gene usage and isotype, and then were expressed as recombinant monoclonal antibodies (mAbs). MAbs were assayed for binding to Env proteins and for neutralization of multiple HIV-1 strains.

## Results

We isolated 13 mAbs that bound Env proteins; of these, 11 were members of three clonal lineages, each of which spanned two time points. Six mAbs in one lineage were all IgG1, used V _H_ 1~69, had an average mutation frequency of 12%, mapped to the CD4 contact region, and had modest neutralization activity. In contrast, three mAbs in a second lineage had two members that were IgA2, used V _H_3~66, had an average mutation frequency of 16%, and neutralized 11/27 (41%) of tier 2 pseudoviruses tested.

## Conclusion

We have isolated the first natural IgA mAbs with broad neutralizing activity. These data demonstrate that class switching to IgA can occur in the generation of bnAbs, an event that is essential for the generation of neutralizing IgA antibodies at mucosal surfaces.

